# Safe Reconstruction of Posterior Sternoclavicular Dislocation Using a Palmaris Longus Autograft and ACL Tibial Guide Assistance: A Case Report

**DOI:** 10.1002/ccr3.72437

**Published:** 2026-04-28

**Authors:** Bilgehan Ocak, Süleyman Kaan Öner, Arda Bilir, Nihat Demirhan Demirkiran

**Affiliations:** ^1^ Department of Orthopaedics and Traumatology Kütahya Health Sciences University Kütahya Turkey

**Keywords:** ACL Tibial guide, figure‐of‐eight reconstruction, open reduction, palmaris longus autograft, posterior sternoclavicular dislocation

## Abstract

Posterior sternoclavicular joint (SCJ) dislocation is an uncommon but clinically significant injury due to its proximity to vital mediastinal structures and delayed or missed diagnosis is not unusual as symptoms may be nonspecific and plain radiographs can appear normal. Surgical stabilization is typically recommended when posterior displacement results in persistent symptoms or a risk to mediastinal anatomy. We report a 32‐year‐old recreational futsal player with posterior SCJ dislocation who presented with shoulder pain, dyspnea, and mild dysphagia following a posterolateral impact, with imaging confirming the diagnosis. Open reduction and figure‐of‐eight reconstruction using a palmaris longus autograft was performed, and tunnel placement was controlled using an anterior cruciate ligament (ACL) tibial guide to reduce the risk of posterior cortical breach and mediastinal injury. Respiratory and swallowing symptoms resolved immediately postoperatively, and at 12 months the patient demonstrated full pain‐free shoulder function and radiographic joint stability without recurrence, suggesting that this technique may offer a safe and durable option for managing posterior SCJ dislocation.

## Key Clinical Message

Use of an ACL tibial guide enabled safe tunnel placement during figure‐of‐eight sternoclavicular reconstruction with a palmaris longus autograft, reducing the risk of mediastinal injury while restoring excellent clinical and functional outcomes.

## Graphical abstract

Use of an ACL tibial guide enables precise and safe tunnel placement during figure‐of‐eight reconstruction for posterior sternoclavicular joint dislocation, reducing the risk of mediastinal injury while restoring joint stability.

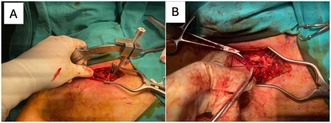

## 1 Introduction

The sternoclavicular joint (SCJ) is the only true bony articulation connecting the upper extremity to the axial skeleton and plays a key role in shoulder girdle stability and motion [1]. Despite strong ligamentous support, its small articular surface makes it inherently unstable and vulnerable to traumatic dislocation. SCJ injuries are rare, accounting for roughly 3% of all shoulder‐girdle dislocations and are classified as anterior or posterior, depending on the direction of displacement [2, 3].

Posterior SCJ dislocation is particularly uncommon, representing only 3%–5% of SCJ dislocations, and often results from high‐energy trauma such as motor vehicle collisions or contact sports [4]. The typical injury mechanism involves a direct blow to the anteromedial clavicle or an indirect posterolateral force applied to the shoulder, resulting in posterior displacement of the medial clavicle [5].

Although rare, posterior SCJ dislocations are associated with significant morbidity due to the proximity of vital mediastinal structures including the trachea, esophagus, subclavian vessels, and brachial plexus [6]. Compression or injury to these structures may cause respiratory, gastrointestinal, or vascular complications, underscoring the need for early diagnosis and prompt reduction [6, 7, 8, 9].

In this report, we describe a rare case of traumatic posterior SCJ dislocation treated with open reduction and figure‐of‐eight reconstruction using a palmaris longus autograft. During the reconstruction, an anterior cruciate ligament (ACL) tibial guide was used to create controlled bone tunnels in the manubrium and clavicle, ensuring precise tunnel placement and protection of the mediastinal structures. This case emphasizes the importance of early diagnosis, the use of tendon autografts with image‐guided instrumentation to achieve stable reconstruction while minimizing mediastinal risk.

## 2 Case History/Examination

A 32‐year‐old right‐hand–dominant recreational futsal player presented to the emergency department after sustaining a fall with a posterolateral impact to the right shoulder. He reported acute pain in the right shoulder girdle, dyspnea, and mild dysphagia. Physical examination demonstrated tenderness over the sternoclavicular region, limited active range of motion of the right shoulder due to pain, and visible depression of the right sternoclavicular joint compared with the contralateral side. Neurovascular examination of the upper extremity was normal. Routine radiographs were initially interpreted as normal, and the patient was discharged. Persistent dyspnea prompted reevaluation four days later at the chest surgery clinic, where sternoclavicular pathology was suspected and orthopedic referral was performed.

## 3 Methods (Differential Diagnosis, Investigations and Treatment)

**FIGURE 1:**
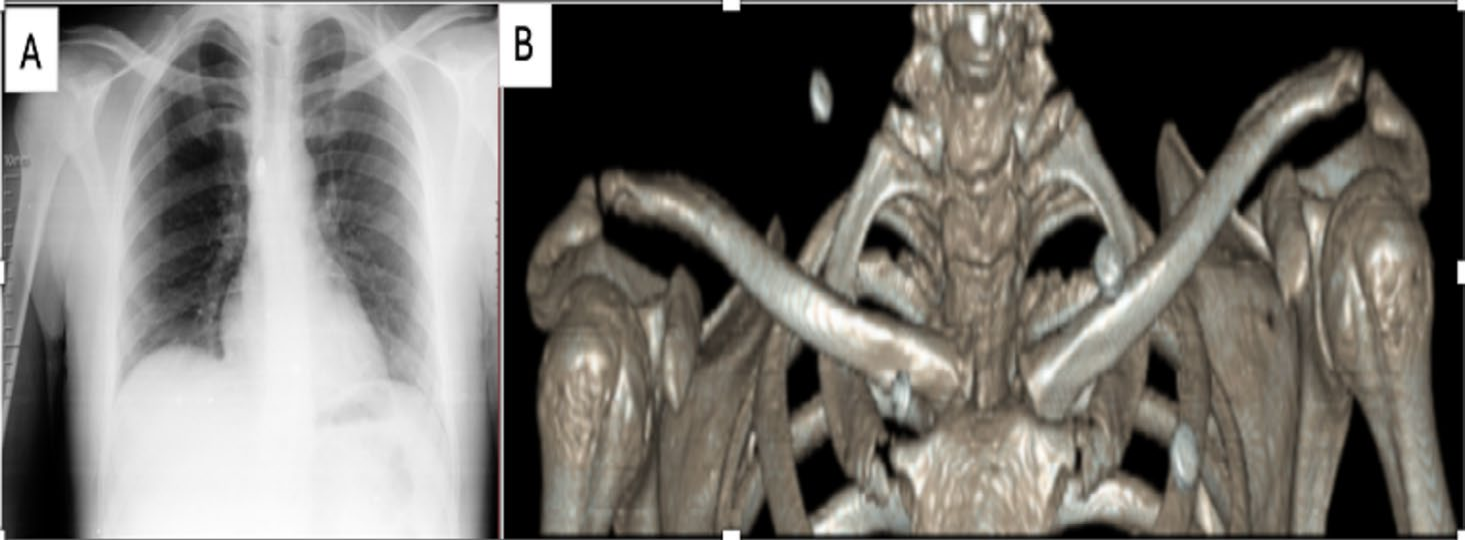
Initial imaging demonstrating posterior sternoclavicular joint dislocation. (A) Anteroposterior chest radiograph showing asymmetry of the medial clavicles with depression of the right sternoclavicular joint, raising suspicion for posterior displacement. (B) Three‐dimensional computed tomography (3D‐CT) confirming posterior sternoclavicular joint dislocation of the right medial clavicle without associated fracture.

Palpation revealed posterior subluxation of the sternoclavicular joint. Repeat anteroposterior radiographs demonstrated asymmetry of the medial clavicle position, and computed tomography (CT) confirmed posterior sternoclavicular joint dislocation without fracture (Figure 1). No evidence of tracheal, esophageal, or vascular compression was identified on imaging; however, the patient's symptoms suggested intermittent mediastinal irritation. Given the risk of further displacement and potential injury to vital mediastinal structures, surgical treatment was planned in coordination with the cardiothoracic surgery team. Under general anesthesia, the patient was positioned supine with a cylindrical bolster placed beneath the interscapular region. Closed reduction was initially attempted but failed, prompting open reduction and reconstruction.

The palmaris longus tendon was first identified clinically at the distal forearm before the initiation of surgery by asking the patient to oppose the thumb and fifth finger while flexing the wrist. A 2‐cm transverse incision was made proximal to the wrist crease, and the antebrachial fascia was incised. The tendon was isolated with blunt dissection and secured with a tendon loop. A tendon stripper was then advanced proximally to release the tendon along its full length, yielding a 16‐cm graft. The harvested tendon was cleaned of residual muscle fibers, tensioned, and prepared for passage through a 4.5‐mm tunnel. Krackow sutures using high‐strength, nonabsorbable, braided polyethylene suture were placed at both ends, and the graft was wrapped in rifampicin‐soaked gauze until implantation.

**FIGURE 2:**
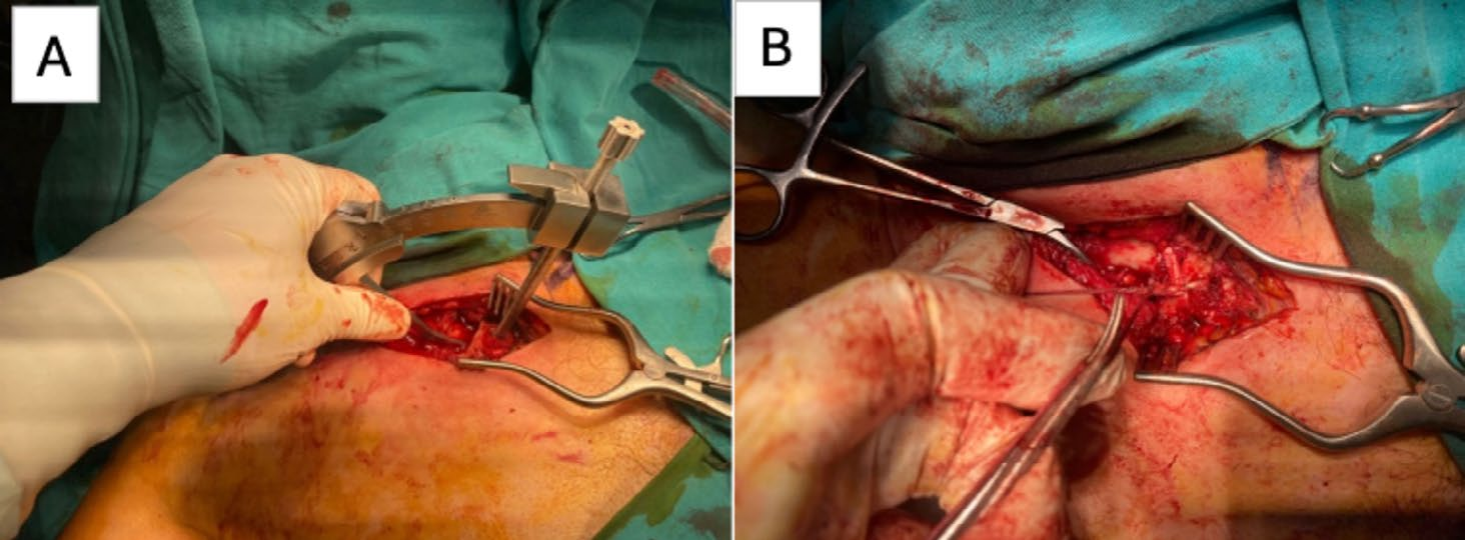
Intraoperative findings and reconstruction technique. (A) Intraoperative exposure of the sternoclavicular joint demonstrating the medial clavicle and manubrium after capsular release. (B) Figure‐of‐eight tendon reconstruction technique with transosseous tunnels in the clavicle and sternum, showing the graft crossing pattern used to restore joint stability.

A transverse 8‐cm incision was made over the right sternoclavicular joint, followed by subperiosteal dissection to expose the joint. After achieving reduction, two 4‐mm tunnels were drilled into the manubrium, approximately 1 cm medial to the articular surface and spaced 1 cm apart. An ACL tibial guide was positioned at approximately 55°–60° relative to the anterior manubrial cortex. Under the controlled guidance of the ACL tibial guide, the drill crossed the posterior cortex, and the guide's depth‐limiting mechanism prevented any further posterior progression. A corresponding posterior–anterior tunnel was then created in the medial clavicle 1 cm lateral to its articular surface. This controlled depth‐stop mechanism enabled safe perforation of the posterior cortex without the need for fluoroscopy and effectively minimized the risk of mediastinal injury. After preparing the bone tunnels, the palmaris longus graft was passed through the clavicular and sternal tunnels in a figure‐of‐eight configuration (Figure 2) and secured with two nonabsorbable Ethibond sutures while maintaining reduction. Postoperative CT confirmed anatomic alignment of the joint. The patient's preoperative respiratory and swallowing difficulties resolved immediately after surgery. A Velpeau bandage was applied for 4 weeks, allowing only pendulum exercises.

## 4 Conclusions and Results (Outcome and Follow‐Up)

Postoperative functional recovery was monitored using the Visual Analog Scale (VAS), Constant Score, American Shoulder and Elbow Surgeons (ASES) score, and the Nottingham Clavicle Score at routine clinical follow‐up visits. All metrics demonstrated a progressive improvement throughout the recovery period. The VAS score decreased from 9 preoperatively to 0 by the third postoperative month, indicating complete resolution of pain. The Constant Score improved from 24 preoperatively to 96 at the final follow‐up, reflecting near‐full restoration of shoulder strength and range of motion. ASES and Nottingham Clavicle Scores showed similar positive trajectories, with marked improvements observed from the first month onward and continued gains through the 12‐month assessment (Table 1). Overall, the radiological confirmation of joint stability combined with the consistent improvements across multiple validated scoring systems demonstrated excellent clinical and functional outcomes following palmaris longus autograft–based figure‐of‐eight reconstruction.

**TABLE 1:** Functional outcome scores at follow‐up visits.

Functional scores	Preoperative	1 month	3 months	6 months	12 months
VAS	9	2	0	0	0
Constant	24	65	82	90	96
ASES	35	70	85	92	97
Nottingham Clavicle	20	68	84	92	98

Abbreviations: ASES, american shoulder and elbow surgeons score; VAS, visual analog scale.

## 5 Discussion

Posterior sternoclavicular joint (SCJ) dislocation is a rare yet clinically serious injury due to the close anatomical relationship between the SCJ and critical mediastinal structures [9]. Delayed or missed diagnosis is not uncommon, as initial symptoms may be nonspecific and standard radiographs may fail to accurately detect posterior displacement [7]. In the present case, the initial misinterpretation of plain radiographs resulted in delayed referral, underscoring the importance of maintaining a high index of suspicion when patients present with shoulder girdle pain accompanied by dyspnea or dysphagia.

Varius treatment strategies have been described to address posterior SCJ dislocation, ranging from closed reduction to various open fixation and reconstruction techniques [3]. Closed reduction may be sufficient for acute and isolated posterior dislocations; however, failure to maintain reduction, persistent instability, or delayed diagnosis often necessitates surgical intervention [10]. Tendon graft–based reconstructions have gained popularity due to their biomechanical strength and ability to restore physiological joint stability without requiring rigid implants that risk migration or injury to mediastinal structures [11].

Several autografts have been used for SCJ stabilization, including semitendinosus, gracilis, and sternocleidomastoid tendons. Biomechanical studies have shown that figure‐of‐eight reconstruction using a semitendinosus graft offers strong initial stability compared with other tendon‐based techniques [11]. However, the palmaris longus autograft—although less frequently reported—provides important advantages, including minimal donor‐site morbidity, adequate graft length, and ease of harvest. In the present case, the palmaris longus graft provided sufficient strength and allowed biological integration for long‐term stability. Compared with more commonly used tendon grafts such as the semitendinosus or gracilis, the palmaris longus offers several practical benefits [12, 13]. Although semitendinosus and gracilis tendons demonstrate high tensile strength in isolated biomechanical testing, their harvest is associated with donor‐site morbidity, including postoperative knee discomfort, flexion weakness [14, 15]. The palmaris longus, in contrast, can be harvested through a small forearm incision with minimal functional deficit and no impact on extremity performance [16]. Its length and diameter are generally suitable for figure‐of‐eight SCJ reconstruction, and existing reports support its capacity for biological incorporation and durable joint stability [11]. These characteristics make the palmaris longus a valuable low‐morbidity autograft option, particularly for young and active patients. In the present case, the palmaris longus graft provided sufficient strength and allowed biologic integration for long‐term stability.

Tunnel placement is a critical component of SCJ reconstruction given the close anatomical relationship to the mediastinum. To enhance safety, an anterior cruciate ligament (ACL) tibial guide was used intraoperatively to control drilling angulation and depth. While some authors recommend shielding the posterior cortex with a raspatory or handheld instrument, this method relies heavily on manual positioning and does not provide consistent trajectory control [13]. In contrast, the ACL tibial guide allows predetermined tunnel orientation and incorporates a depth‐limiting mechanism that reduces the risk of excessive posterior penetration. Although not widely described in the SCJ literature, this technique may serve as a valuable adjunct to improve safety during tunnel creation.

The postoperative clinical outcomes in this case were excellent, with full pain relief, restoration of shoulder strength and range of motion, and radiographic confirmation of stability at 12 months. Improvements across VAS, Constant Score, ASES, and Nottingham Clavicle Score further support the efficacy of palmaris longus autograft–based figure‐of‐eight reconstruction in restoring joint stability and shoulder function following posterior SCJ dislocation.

Posterior sternoclavicular joint dislocation is an uncommon but potentially life‐threatening injury due to the proximity of critical mediastinal structures. Early recognition and appropriate management are therefore essential to prevent catastrophic complications. In this case, open reduction and figure‐of‐eight reconstruction with a palmaris longus autograft resulted in complete pain relief, restored range of motion, and stable joint alignment at long‐term follow‐up. The use of an ACL tibial guide during tunnel creation provided controlled drill angulation and improved safety by minimizing the risk of posterior cortical breach and mediastinal injury. This report highlights tendon autograft–based reconstruction—supported by image‐guided instrumentation—as a safe and effective surgical option for achieving durable stability and excellent functional outcomes in posterior SCJ dislocation.

## Author Contributions

**Bilgehan Ocak:** conceptualization, data curation, formal analysis, investigation, methodology, project administration, resources, software, supervision, validation, visualization, writing – original draft, writing – review and editing. **Süleyman Kaan Öner:** conceptualization, data curation, investigation, methodology, visualization, writing – original draft, writing – review and editing. **Arda Bilir:** conceptualization, data curation, investigation, methodology, project administration, software, visualization, writing – original draft, writing – review and editing. **Nihat Demirhan Demirkiran:** conceptualization, data curation, formal analysis, investigation, methodology, project administration, resources, software, supervision, validation, visualization, writing – original draft, writing – review and editing.

## Funding

The authors have nothing to report.

## Ethics Statement

Ethical approval was not required for this single‐patient case report in accordance with institutional policy.

## Consent

Written informed consent was obtained from the patient for publication of clinical information and images.

## Conflicts of Interest

The authors declare no conflicts of interest.

## Data Availability

Data supporting this case report are available from the corresponding author upon reasonable request.
